# Structural and mechanistic perspectives on Nse5/6 regulation of the Smc5/6 complex

**DOI:** 10.3389/fmolb.2026.1757821

**Published:** 2026-04-29

**Authors:** Ji-Hyun Kim, Dharanikota Rishita Rao, Kyoung-Dong Kim

**Affiliations:** Department of Systems Biotechnology, Chung-Ang University, Anseong, Republic of Korea

**Keywords:** ATPase regulation, chromatin association, genome stability, Nse5/6 heterodimer, Smc5/6 complex

## Abstract

The Smc5/6 complex is a vital protector of eukaryotic genome stability, coordinating DNA repair, replication fork maintenance, recombination intermediate processing, and chromosome organization. Within this complex, the Nse5/6 heterodimer has recently emerged as a key factor influencing Smc5/6 dynamics, acting at the interface of structural control, enzymatic regulation, and chromatin recruitment. Structural studies from yeast to mammals show that Nse5/6 associate with the Smc5/6 head–neck region, restricting ATPase head engagement and stabilizing an inactive, chromatin-loading-ready state. Upon ATP binding and DNA interaction, conformational changes displace or reposition Nse5/6, facilitating Nse4-mediated head closure, DNA entrapment, and loop-modulating activity. Functional analyses across *Saccharomyces cerevisiae*, *Schizosaccharomyces pombe*, mammals, and plants indicate that Nse5/6 is essential for recruiting Smc5/6 to damaged or stalled replication forks, stabilizing chromatin association, and coordinating SUMO-dependent repair pathways. Loss of Nse5/6 leads to defects in replication stress tolerance, accumulation of recombination intermediates, impaired chromatin loading, and widespread genome instability. This review synthesizes emerging structural and functional insights into Nse5/6, emphasizing its conserved yet species-adapted mechanisms that regulate ATPase gating, DNA substrate selection, and chromatin recruitment. Collectively, these findings redefine Nse5/6 not as a peripheral structural factor but as a dynamic regulatory hub that orchestrates Smc5/6 activity in genome maintenance, development, and antiviral defense.

## 1 Introduction

The Structural Maintenance of Chromosomes (SMC) 5/6 complex is a highly conserved protein complex that is composed of several subunits, including Smc5, Smc6, along with the non-SMC subunits Nse1, Nse2 (Mms21), Nse3, Nse4, Nse5, and Nse6. Each NSE subunit collaborates with others to create distinct submodules within the SMC5/6. This complex plays a crucial role in maintaining genome stability by facilitating sister chromatid cohesion (Potts et al., 2006; Jeppsson et al., 2014; Stephan et al., 2011), resolving recombination intermediates during DNA repair (Copsey et al., 2013; Atkins et al., 2020), and ensuring genome maintenance associated with transcription (Diman et al., 2024; Roy et al., 2024; Wu et al., 2026).

Compared to other SMC complexes like cohesin and condensin, Smc5/6 is less well defined in terms of structure and mechanism. However, recent structural and biochemical studies has shed light on the specific functions of the Nse5/6 submodule within the SMC5/6 complex. Importantly, the Nse5/6 subunits act as a regulatory hub that influences the complex’s ATPase activity and aids in dynamic interactions related to chromatin recruitment and DNA repair (Taschner et al., 2021).

In this review, we will discuss the emerging roles of the Nse5/6 sub complex as a dynamic regulator of the Smc5/6 complex across eukaryotic species. By integrating structural flexibility with diverse binding partners, Nse5/6 modulates the spatial and temporal control of Smc5/6 activities during genome surveillance and stress responses. We will highlight recent advances that clarify their species-specific roles in replication stress responses, homologous recombination repair, and broader chromatin organization, as well as their conserved and specialized functions from yeast to mammals and plants. These insights collectively underscore Nse5/6 as indispensable mediators of genome maintenance, whose biological significance is only beginning to be fully appreciated.

## 2 Architecture and positioning of Nse5/6 within the SMC5/6 complex

**FIGURE 1:**
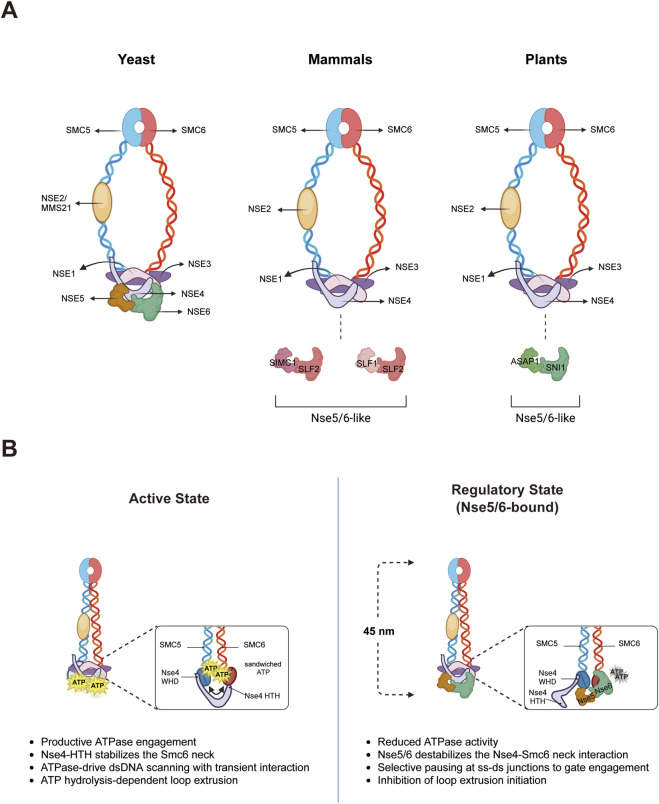
Structure and conformational regulation of the SMC5/6 complex by Nse5/6. **(A)** Structure of the SMC5/6 complex in yeast, mammals, and plants based on the currently available data. The SMC5/6 core complex (SMC5/6 and NSE1-4) is highly conserved across eukaryotes, whereas the NSE5/6 submodule exhibits evolutionary divergence. In mammals and plants, functionally analogous Nse5/6-like equivalents (SIMC1/SLF1-SLF2 and ASAP1-SNI1, respectively) serve this function. **(B)** Conformational states and ATPase regulation of the SMC5/6 complex by Nse5/6. In the active state, Nse4 interacts with SMC6 to promote productive ATPase engagement. This state drives transient, ATPase-dependent dsDNA scanning and ATP-dependent DNA loop extrusion (left). In the Nse5/6-bound regulatory state, Nse5/6 destabilizes the Nse4-SMC6 interaction. This structural rearrangement reduces ATPase activity, potentiating selective pausing at ss-dsDNA junctions to gate engagement and inhibit the initiation of loop extrusion (right). This figure was created with BioRender.com.

The SMC5/6 complex is characterized by a shared trimeric core structure found in all SMC complexes, comprising an SMC dimer and kleisin. However, it is unique due to its specific regulatory subunits like KITE and additional enzymatic roles beyond ATPase, such as the Nse2 SUMO ligase and Nse1 ubiquitin ligase. Structurally, the complex is divided in three main modules: Smc5-Smc6-Nse2/Mms21, Nse1-Nse3-Nse4, and Nse5-Nse6 (Figure 1A) (Duan et al., 2009a). Each of these submodules has a distinct function in the Smc5/6 complex, especially in DNA repair and chromosome maintenance.

The Smc5-Smc6-Nse2/Mms21 core consists of Smc5 and Smc6 forming a hinge-mediated heterodimer. This heterodimer, organized into hinge, arm, and head domain, acts as a structural base for ATP-dependent DNA interaction. Within this heterodimer, Nse2 is directly attached to the mid-arm region of Smc5 (Duan et al., 2009b; Varejão et al., 2018). The SP-RING domain of Nse2 serves as the catalytic center for SUMO E3 ligase activity, with SUMO-interacting motifs (SIMs) aiding in SUMO engagement (Varejão et al., 2021). This setup allows Nse2 to SUMOylate internal Smc5/6 subunits (Smc5, Smc6 and Nse4) (Varejão et al., 2018; Andrews et al., 2005; Bermúdez-López et al., 2015) as well as external targets like the Sgs1-Top3-Rmi1 complex (Bermúdez-López et al., 2016), thereby enhancing complex stability and facilitating DNA repair and recombination processes.

The Nse1-Nse3-Nse4 module is located at the head-proximal coiled coil (neck) of the Smc5-Smc6 heterodimer, aiding in DNA interaction. At the core of this configuration is the kleisin subunit Nse4, which connects its N-terminal HTH domain to the Smc6 neck and its C-terminal WHD to the Smc5 head, thus enabling the closure of the SMC ring. Associated with the kleisin subunit is the KITE dimer composed of Nse1 and Nse3. Nse3 directly interacts with Nse4 through a conserved hydrophobic surface in its C-terminal domain, while its N-terminal region mediates the interaction with Nse1 (Torres-Rosell et al., 2007; Hudson et al., 2011; Guerineau et al., 2012; Jo et al., 2021). Nse1 features a RING-like domain that contributes to the complex’s function. Nse3 provides a double-stranded DNA binding surface that aids in recruiting the Smc5/6 complex to highly repetitive telomeric and ribosomal DNA sites (Pebernard et al., 2008; Moradi-Fard et al., 2016; Zabrady et al., 2016; Moradi-Fard et al., 2021). This arrangement supports ATPase coordination and the DNA damage response.

Lastly, Nse5-Nse6 form a helical-repeat heterodimer that attaches near the ATPase head region of Smc5/6 (Yu et al., 2021). Both Nse5 and Nse6 directly interact with Smc6, with Nse6 specifically targeting the neck regions of Smc6 through its C-terminal half. This interface between Nse6 and Smc6 is close to where Nse4 binds the Smc6 neck (Li et al., 2023). These interfaces can be subtly adjusted based on ATP and DNA substrate. Despite having weak sequence homology (Pebernard et al., 2006; Taylor et al., 2008; Watanabe et al., 2009; Hallett et al., 2021), their structural and functional roles are evolutionarily conserved. Interestingly, while Nse5/6 is critical for viability in most other organisms, it is not essential in fission yeast (Pebernard et al., 2006). Historically, their positioning was debated due to conflicting reports across different yeast species. Research in *S. pombe* indicated a head-proximal localization, whereas studies in *S. cerevisiae* suggested a hinge-proximal position, supported by *in vitro* pull-down and two-hybrid experiments (Duan et al., 2009a; Palecek et al., 2006). However, recent high-resolution structural analyses favor a predominantly head-proximal location for Nse5-Nse6 (Li et al., 2023; Hallett et al., 2021). In this position, Nse5-Nse6 physically interacts with the Smc6, either regulating ATPase activity or promoting the formation of SMC ring to aid in DNA engagement. This regulatory framework is extended to higher eukaryotes through functional equivalents. In humans, SLF1 and SLF2 have been recognized as analogous to Nse5/6-like domain of yeast Nse5 and Nse6, respectively (Figure 1A) (Räschle et al., 2015). These human proteins associate with the SMC5/6 complex, influencing its roles in restricting viral DNA and maintaining replication fork stability. In plants, the counterparts to Nse5/6 are ASAP1 and SNI1, which form a plant-specific heterodimer similar to fungal Nse5-Nse6 (Figure 1A). ASAP1 connects subunits allows the SMC5/6 core with SNI1, facilitating DNA repair, genome stability, and the regulation of defense genes in *Arabidopsis thaliana* (Zhu et al., 2021).

The dynamic interplay between these subunits allows the SMC5/6 complex to assume various configurations, enabling its involvement in a broad spectrum of cellular activities. This structural complexity is crucial for the diverse functions associated with the SMC5/6 complex, including its critical roles in DNA repair, chromosome maintenance, and other chromosomal processes.

## 3 Nse5/6-mediated control of Smc5/6 conformational dynamics, ATPase activity, and DNA engagement

SMC5/6 complexes experience notable conformational changes as they switch between ATP-free and ATP-bound states, affecting their DNA-binding capabilities and allowing for precise control over their roles in chromosome organization, repair, and segregation. Central to this regulation is the shift from an rod-shaped conformation in the ATP-free (apo) state to an engaged state when ATP is bound (Taschner et al., 2021; Hallett et al., 2022). Although this cycle is essential for all SMC complexes, the Nse5/6 heterodimer plays a pivotal role by inducing additional rearrangements, potentially stabilizing or locking the complex in specific functional states (Taschner et al., 2021; Li et al., 2023; Hallett et al., 2021). Consequently, the modulation of ATPase activity by Nse5-Nse6 has become a key area of research to understand how the complex performs advanced functions like substrate-specific genome maintenance and loop extrusion.

The understanding of Nse5/6 function has evolved from an initial concept of a ‘physical blocker’ to a more nuanced regulatory mechanism. Early biochemical and structural studies identified Nse5/6 as a negative regulator that inhibits the enzymatic core of the entire complex. Specifically, the presence of Nse5/6 in the Smc5/6 hexamer was shown to cause strong, DNA-independent suppression of ATPase activity. Negative-strain EM and structure modeling indicated that Nse5/6 is located near the head region, where it exerts hindrance that physically prevents head engagement and ATP turnover (Hallett et al., 2021). Additional evidence from XL-MS (Cross-Linking Mass) and cysteine cross-linking suggested that Nse5-Nse6 anchors the ATPase head by positioning itself across the arm and head-arm junction, effectively keeping the complex in an inhibitory state that is only partially relieved upon ATP binding and interaction with specific DNA substrates (Taschner et al., 2021). Nse5/6 also contributes to reducing the overall length of the complex about 45 nm by binding to the head-end of the SMC5/6 complex, causing a significant conformational change. This structural modification is likely essential for the complex’s functionality (Figure 1B) (Hallett et al., 2021).

Recent high-resolution cryo-EM studies have enhanced this model, moving the focus from mere spatial inhibition to a dynamic “neck-gate” regulatory mechanism (Li et al., 2024). This model proposes that Nse5-Nse6 are not just passive inhibitors but actively compete with the neck region of Smc6 and the Nse1-Nse3-Nse4 submodule. Typically, the interaction between the Nse4-HTH domain and the Smc6 neck stabilizes the Nse1-Nse3-Nse4 submodule, maintaining the structural integrity of the apo state and enabling the transition to an active state upon ATP binding. However, when Nse5/6 binds, Nse6 effectively displaces Nse4 from the Smc6 neck, causing the Nse1-Nse3-Nse4 submodule to adopt a more flexible and less stable configuration (Li et al., 2023). This displacement does not completely prevent head engagement but rather destabilizes the “neck-gate”, leading to an inhibitory state with significantly reduced ATPase efficiency and altered DNA engagement modes. Through this competitive regulation, Nse5/6 can precisely control the ATPase head’s binding, acting as a sophisticated switch that determines the SMC5/6 complex’s functional mode in response to various genomic contexts (Figure 1B).

The importance of structural rearrangement goes beyond merely ATPase inhibition. Instead, the conformational changes prompted by Nse5/6 can be seen as leading to a functional outcome that dictates the conditions under which the SMC5/6 complex’s inherent DNA processing activity is effectively utilized. This is achieved by determining the timing and stability of Smc5/6’s interaction with a specific DNA substrate (Taschner et al., 2021; Li et al., 2023). Understanding the DNA-binding and reorganization abilities of the SMC5/6 holo-complex is crucial. The yeast SMC5/6 holo-complex (Nse5/6-free hexamer) naturally displays DNA-dependent ATP hydrolysis and Nse2-dependent SUMO E3 ligase activity. It is recognized as a complex capable of ATP hydrolysis-dependent topological DNA binding, preferential binding to supercoiled and catenated DNA, stabilization of DNA plectonemes, and performing low-speed ATP-dependent DNA compaction (Gutierrez-Escribano et al., 2020; Kanno et al., 2015). Thus, SMC5/6 is not merely a DNA binding protein; it has essential properties that allow it to identify and stabilize the tertiary structure of DNA formed by closely aligned DNA helices (Gutierrez-Escribano et al., 2020). Building on the inherent DNA-processing abilities of this holo-complex, yeast Nse5/6 seems to act as a higher regulatory layer that imposes ATPase restraint and substrate discrimination (Taschner et al., 2021; Hallett et al., 2021; Oravcová et al., 2022). Although Nse5/6 is suggested to function similarly to the Scc2-Scc4 cohesin loader complex, there are significant differences. Unlike Scc2-Scc4, which enhances ATPase activity and directly binds to DNA, Nse5/6 inhibits ATPase activity and does not have intrinsic DNA-binding capability (Hallett et al., 2021).

Nse5/6 employs competitive state transitions during the recognition of DNA substrates to connect the length of the inhibition phase with substrate selectivity. The octamer with Nse5/6 diffuses on dsDNA and stably accumulates at ssDNA-dsDNA junctions (Gutierrez-Escribano et al., 2020; Tanasie et al., 2022), whereas the hexamer without Nse5/6 exhibits the opposite DNA-substrate preference in ATPase assays, with short linear DNA being more stimulatory than plasmid DNA (Taschner et al., 2021). Additionally, single-molecule studies reveal that the Nse5/6-free hexamer tends to frequently explore junctions with short dwell times, while the Nse5/6-bound octamer decreases contact frequency and increases dwell time, thus managing the shift between ss-ds junction stabilization and dsDNA exploration (Taschner and Gruber, 2023; Oravcová et al., 2025). These findings support the model where the Nse5/6 neck gate controls the positioning of Nse1-Nse3-Nse4 to facilitate effective topological bridging only when substrate compatibility is confirmed (Oravcová et al., 2022; Taschner and Gruber, 2023; Oravcová et al., 2025). This idea offers a framework that can be applied to the human SMC5/6. Given that the human SMC5/6 holo-complex also has structure-specific DNA-binding and ATP-dependent compaction activity, Nse5/6 likely acts as a regulatory module that fine-tunes the holo-complex’s core activity, rather than providing new DNA-binding abilities, to enable selective entrapment and fork-protective bridging on appropriate DNA substrates (Serrano et al., 2020). Overall, Nse5/6 serves as a ‘molecular toggle’, intricately coordinating the complex’s spatial and temporal interaction with various DNA structures (Chang et al., 2026).

Recent single-molecule imaging research has significantly broadened the understanding of SMC5/6 complex, highlighting its role as a loop extruder, similar to other SMC complexes, beyond its involvement in DNA substrate selection and structural stabilization. In the loop extrusion process of SMC5/6 complex, variations such as symmetry, asymmetry, and conformational states are still actively debated (Pradhan et al., 2023; Barth et al., 2025). These studies have revealed that the Nse5/6 module serves as a negative regulator of loop extrusion, influencing the formation of dimeric Smc5/6 for symmetrical two-sided DNA reeling. Interestingly, the presence of Nse5/6 greatly decreases the likelihood of loop initiation and Smc5/6 dimerization, yet it does not affect the rate or dwell time after loop formation (Pradhan et al., 2023). This indicates that Nse5/6 does not disrupt the ongoing extrusion kinetics but rather plays a regulatory role during the initiation phase, possibly by controlling the ATPase-dependent structural transition or dimerization state necessary to start the extrusion process.

## 4 Role of Nse5/6 complex in genome maintenance under replication stress

**FIGURE 2:**
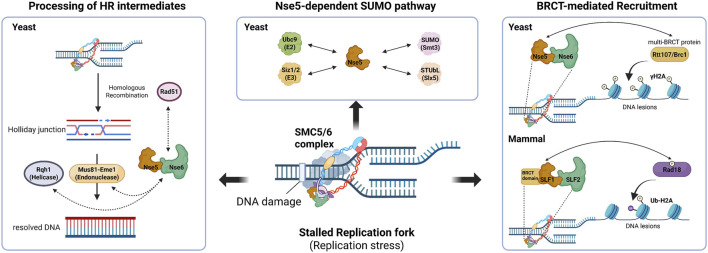
Nse5/6-mediated regulation of SMC5/6 at stalled replication fork and DNA Processing of HR intermediates via helicase/endonuclease interactions. Nse5/6 promotes the resolution of junction DNA intermediates during homologous recombination, in concert with endonucleases and helicases. Consistently, nse5/6 mutants show rad51Δ-suppressible UV sensitivity (Left). Nse5 functions as a SUMO pathway hub. Nes5 directly interaction SUMO-related factors to facilitate Nse2-depedent SUMOylation of the SMC5/6 (Center). Nse5/6 facilitate SMC5/6 recruitment to DNA lesions via interplay with BRCT. In yeast, multi-BRCT proteins bind to γH2A and promote recruitment of Nse5/6-SMC5/6 to DNA damage sites. In mammals, the SLF1-SLF2 is recruited to stalled replication fork through phosphor-RAD18 recognition by the SLF1 BRCT domain (Right). This figure was created with BioRender.com.

The SMC5/6 complex plays a crucial role in preserving the integrity of stalled or collapsed replication. The structural and enzymatic significance of the Nse5/6 submodule is likely connected to SMC5/6 functions through interactions with specific factors. Research suggests that Nse5/6 serves as a link between the core Smc5/6 and other regulatory proteins, including SUMO ligases, helicases, endonucleases, and DNA damage response factors, thereby coordinating various DNA repair pathways (Figure 2).

In yeast, Nse5/6 facilitates Nse2-dependent SUMOylation of replisome and repair factors, such as kleisin Nse4 (Varejão et al., 2018; Yu et al., 2021; Bustard et al., 2016). During replication stress, Nse5/6 recruits SMC5/6 to Nse2 at the stalled fork, enhancing the SUMOylation of Nse4 and other factors to stabilize the replisome and restart the fork (Pebernard et al., 2006; Irmisch et al., 2009; Bustard et al., 2012; Oravcová et al., 2019). Additionally, in budding yeast, components of the SUMO pathway, such as Smt3, the E2 enzyme Ubc9, and the PIAS family E3 ligases Siz1 and Siz2, interact with Nse5 (Bustard et al., 2016; Bustard et al., 2012), and Nse5 also partially aids in the recruitment and stabilization of the SUMO-targeted ubiquitin ligase subunit Slx5 (Horigome et al., 2016). In fission yeast, the interaction between Rfp1 (the functional equivalent of Slx5) and Nse5 was confirmed through yeast two-hybrid screening (Prudden et al., 2007). Thus, the inactivation of these SUMO factors leads to decreased SMC5/6-associated SUMOylation, highlighting the role of Nse5/6 in SUMO assembly during the replication stress response.

Nse5/6 plays a crucial role in facilitating interactions with helicases and structure-specific endonucleases to manage the processing of DNA intermediates that form during replication stress. When replication stress occurs, stalled replication forks are frequently handled through homologous recombination, a mechanism that produces junction molecules (JMs), such as Holliday junctions (HJs). Nse5/6 is vital for preventing the buildup of these HR intermediates. In fission yeast, the UV sensitivity of *nse5*/*6* mutants is entirely suppressed by the deletion of Rad51 (Pebernard et al., 2006). Additionally, a deficiency in *nse5*/*6* results in synthetic lethality with the HJ resolvase subunit Mus81 and the RecQ type DNA helicase Rqh1 (Pebernard et al., 2006). Although not related to replication stress, a similar process takes place during meiosis. *nse6Δ* accumulates JMs in a manner similar to *mus81Δ*, indicating that the Mus81-Eme1 endonuclease is aided by Nse5/6 in resolving Rec12-dependent single HJ (Wehrkamp-Richter et al., 2012). In budding yeast, the DNA helicase Mph1 directly associates with Smc5 through yeast two-hybrid, co-IP, and pull-down assays, and is immunoprecipitated with Nse6 (Chen et al., 2009). However, the genetic interaction between Nse5/6 and Mph1 remains uncertain.

Additionally, Nse5/6 is essential for detecting genomic damage and physically brings the SMC5/6 complex to damaged DNA sites by interacting with DNA damage response factors. In yeast, when DNA damage occurs, Nse5/6 associates with multi-BRCT proteins Rtt107 (*S. cerevisiae* (Leung et al., 2011; Wan et al., 2019)/Brc1 (*S. pombe*) (Oravcová et al., 2019; Etheridge et al., 2021), which identify phosphorylated histone variants (γH2A) at DNA damage sites to anchor SMC5/6 to stalled replication forks. Furthermore, live-cell single particle tracking (SPT) demonstrated that Nse5/6 directly influences the chromatin association of SMC5/6 in a Brc1-dependent manner in fission yeast (Etheridge et al., 2021). The absence of brc1 decreases SMC5/6 chromatin association similarly to *nse6* deletion, though the effect is less pronounced than with *nse6* deletion, suggesting that SMC5/6 association is mediated through both Brc1-independent and Brc1-dependent pathways. This recruitment mechanism is evolutionarily conserved. In mammals, there are two distinct Nse5-like paralogues: SLF1, which has a BRCT domain repeat at the N-terminals (Huang et al., 2023), and SIMC1, which features an Nse5-like domain and SUMO interaction motifs (Oravcová et al., 2022; Sun and Hunter, 2012). Both form a complex with SLF2 independently. The SLF1-SLF2 complex recognizes phosphorylated serine on the C-terminus of the ubiquitin ligase Rad18 and directly recruits SMC5/6 to stalled replication forks and chromosomal DNA lesions (Räschle et al., 2015; Ryder et al., 2024). Conversely, the SIMC1-SLF2 complex uses its SIMs to bind the SUMO network, directing SMC5/6 to extrachromosomal circular DNA such as viral genomes or plasmids (Oravcová et al., 2022; Oravcová et al., 2025; Sun and Hunter, 2012).

Overall, these findings suggest that Nse5/6 acts as a central regulator that facilitates the recruitment and functional engagement of SMC5/6 complex at DNA damage sites and replication-associated recombination intermediates. Nse5/6 aids in stabilizing the replication fork and resolving intermediates by enabling Nse2-dependent SUMOylation and coordinating interactions with various factors, thereby maintaining genome stability under replication stress.

## 5 Consequences of Nse5/6 dysfunction

The removal or malfunction of the Nse5/6 submodule closely resembles the mutant phenotypes observed in other SMC5/6 subunits. Since Nse5 and Nse6 create a structurally and functionally dependent heterodimer, the absence of either subunit effectively disrupts the regulation of the Nse5/6 module and significantly impairs the function of the entire SMC5/6 complex. The repercussions of this dysfunction are severe and consistently observed across eukaryotes, leading to increased sensitivity to genotoxic stress and genome instability.

In yeast, phenotypic studies of *nse5* and *nse6* mutants highlight the essential nature of this submodule. In *S. pombe*, where both genes are not essential for survival, *nse5Δ* and *nse6Δ* mutants exhibit nearly identical, extreme sensitivity to DNA-damaging agents like UV, methylmethanesulfonate (MMS), and hydroxyurea (HU) (Pebernard et al., 2006; Hayles et al., 2013). Importantly, *nse5Δnse6Δ* double mutants do not show additional hypersensitivity compared to either single mutant, confirming that Nse5 and Nse6 operate as a single interdependent unit (Khan et al., 2022). During mitosis, *nse6Δ* accumulates spontaneous DNA damage, which is exacerbated by UV exposure and often results in mitotic failure, including DNA stretching (Pebernard et al., 2006). In meiosis, *nse6Δ* mutants are unable to resolve recombination intermediates, causing defective chromosome segregation, non-disjunction, and a significant decrease in spore viability (Wehrkamp-Richter et al., 2012). Similarly, in *S. cerevisiae*, *nse5-ts1*, a mutant where the interaction between Nse5 and Nse6 is compromised, shows a severe buildup of homologous recombination intermediates and heightened sensitivity to HU (Bustard et al., 2016; Bustard et al., 2012). Consistent with these observations, the deletion of KRE29p (Nse6 ortholog) in the pathogenic yeast *Candida glabrata* results in reduced cell viability and marked hypersensitivity to UV and MMS (Table 1) (Miyazaki et al., 2006).

**TABLE 1:** Summary of the consequences of Nse5/6 dysfunction.

Species/Model	Defective factor	Main phenotype/ Consequence	Biological significance	Ref
*S. pombe*	nse5Δ, nse6Δ	High sensitivity to UV, MMS, HU	Nse5/6 functions as a single unit	Pebernard et al. (2006), Hayles et al. (2013)
	nse5Δnse6Δ double mutant	No additive phenotype	Functional interdependence of Nse5 and Nse6	Khan et al. (2022)
	*nse6Δ (mitosis)*	DNA damage accumulation, mitotic failure	Required for genome stability during mitosis	Pebernard et al. (2006)
	*nse6Δ (meiosis)*	Defective recombination resolution, chromosome missegregation	Essential for meiotic recombination control	Wehrkamp-Richter et al. (2012)
*S. cerevisiae*	nse5-ts1	HR intermediate accumulation, HU sensitivity	Nse5–Nse6 interaction is critical under replication stress	Bustard et al. (2016), Bustard et al. (2012)
*C. glabrata*	KRE29p deletion (Nse6 ortholog)	Reduced viability, UV/MMS sensitivity	Evolutionarily conserved Nse6 function	Miyazaki et al. (2006)
*P. patens*	Ppnse6 mutant	DNA damage sensitivity, growth defects	Role in DNA repair and development	Lelkes et al. (2023)
	Ppnse5 KO	DNA repair defects	Nse5 contributes to repair pathways	Vaculíková et al. (2024)
*A. thaliana*	SNI1 loss (Nse6 homolog)	DNA damage, growth defects	Genome maintenance and developmental regulation	(Yan et al., 2013; Zhu et al., 2021)
Human (Atelís syndrome)	biallelic SLF2 mutations	Chromosomal instability, replication stress	Linked to developmental disorders	Grange et al. (2022)
Human (DLBCL)	reduced SLF2 expression	Checkpoint defects, genome instability	Associated with cancer progression	Zhang et al. (2023)

This defect’s evolutionary conservation is also observed in plant models. In the moss *Physcomitrium patens*, *Ppnse6* mutant lines remain viable but show sensitivity to the DNA-damaging agent bleomycin, along with a decrease in rDNA copies, stunted growth, and developmental issues (Lelkes et al., 2023). The *Ppnse5* KO mutant line displays DNA repair deficiencies while maintaining a normal count of rDNA repeats (Vaculíková et al., 2024). In *Arabidopsis thaliana*, the absence of SNI1 (Nse6 homologue) leads to spontaneous DNA damage, altered meiotic crossover distribution, and reduced growth (Table 1) (Zhu et al., 2021; Yan et al., 2013).

Importantly, the malfunction of the Nse5/6 module in humans is closely linked to serious clinical disorders. In Atelís syndrome, biallelic mutations in SLF2 (a subunit similar to Nse6) are detected. Cells derived from patients exhibit a distinct chromosomal instability phenotype, spontaneous stalling of replication forks, and a loss of sister chromatid cohesion (Grange et al., 2022). Furthermore, in individuals with diffuse large B-cell lymphoma (DLBCL), there is a reduction in SLF2 expression. The absence of SLF2 hinders the activation of the CHK1-ATR checkpoint in response to DNA damage, resulting in genome instability and facilitating the development of B-cell lymphoma (Table 1) (Zhang et al., 2023).

To sum up, the results indicate that the Nse5/6 submodule is a fundamental element of genome stability in the SMC5/6 complex, rather than just a supplementary part. Without a functional Nse5/6 module, the SMC5/6 complex cannot operate correctly, significantly jeopardizing genome stability. This could result in cell death in lower eukaryotes and lead to serious developmental issues or cancerous diseases in humans.

## 6 Conclusion and perspectives

Collectively, current evidence supports a model in which Nse5 and Nse6 function as multifunctional regulatory subunits that integrate structural organization, ATPase control, DNA substrate selection, and context-dependent recruitment of the Smc5/6 complex. Across eukaryotic systems, these activities position Nse5/6 as key determinants of how Smc5/6 is deployed during DNA repair, replication-associated stress responses, chromatin regulation, development, and antiviral defense. Their functional conservation from yeast to humans underscores that Nse5/6 is not a peripheral accessory module, but a central regulatory interface that coordinates the spatial and temporal control of Smc5/6 activity. Future studies will be needed to define how these regulatory mechanisms are adapted in different species and chromatin contexts, and how their dysregulation contributes to human disease.
